# New insights into neuropathology and pathogenesis of autoimmune glial fibrillary acidic protein meningoencephalomyelitis

**DOI:** 10.1007/s00401-023-02678-7

**Published:** 2024-02-03

**Authors:** Yong Guo, Verena Endmayr, Anastasia Zekeridou, Andrew McKeon, Frank Leypoldt, Katharina Hess, Alicja Kalinowska-Lyszczarz, Andrea Klang, Akos Pakozdy, Elisabeth Höftberger, Simon Hametner, Carmen Haider, Désirée De Simoni, Sönke Peters, Ellen Gelpi, Christoph Röcken, Stefan Oberndorfer, Hans Lassmann, Claudia F. Lucchinetti, Romana Höftberger

**Affiliations:** 1 Department of Neurology, Mayo Clinic, Rochester, MN 55905 USA; 2 Division of Neuropathology and Neurochemistry, Department of Neurology, Medical University of Vienna, Waehringer Guertel 18-20, 1090 Vienna, Austria; 3 Comprehensive Center for Clinical Neurosciences and Mental Health, Medical University of Vienna, Waehringer Guertel 18-20, 1090 Vienna, Austria; 4 Laboratory Medicine and Pathology, Mayo Clinic College of Medicine, Rochester, MN USA; 5 Institute of Clinical Chemistry, University Medical Center Schleswig-Holstein Kiel, Lübeck, Germany; 6 Department of Neurology, University Medical Center Schleswig-Holstein and Kiel University, Kiel, Germany; 7 Institute of Neuropathology, University Hospital Muenster, Muenster, North Rhine Westphalia Germany; 8 Department of Pathology, University Medical Center Schleswig-Holstein, Kiel, Germany; 9 Department of Neurology, Division of Neurochemistry and Neuropathology, Poznan University of Medical Sciences, Poznań, Poland; 10 Institute of Pathology, University of Veterinary Medicine, Vienna, Austria; 11 Internal Medicine, University Clinic for Small Animals, University of Veterinary Medicine, Vienna, Austria; 12 Division of Neurology, Karl Landsteiner University of Health Sciences, University Hospital, St. Pölten, Austria; 13 Clinic for Radiology and Neuroradiology, University Hospital Schleswig-Holstein Campus Kiel, Kiel, Germany; 14 Center for Brain Research, Medical University of Vienna, Vienna, Austria

**Keywords:** GFAP, Autoimmunity, Biopsies, Autopsies, Magnetic resonance imaging

## Abstract

Anti-glial fibrillary acidic protein (GFAP) meningoencephalomyelitis (autoimmune GFAP astrocytopathy) is a new autoimmune central nervous system (CNS) disease diagnosable by the presence of anti-GFAP autoantibodies in the cerebrospinal fluid and presents as meningoencephalomyelitis in the majority of patients. Only few neuropathological reports are available and little is known about the pathogenic mechanisms. We performed a histopathological study of two autopsies and nine CNS biopsies of patients with anti-GFAP autoantibodies and found predominantly a lymphocytic and in one autopsy case a granulomatous inflammatory phenotype. Inflammatory infiltrates were composed of B and T cells, including tissue-resident memory T cells. Although obvious astrocytic damage was absent in the GFAP-staining, we found cytotoxic T cell-mediated reactions reflected by the presence of CD8^+^/perforin^+^/granzyme A/B^+^ cells, polarized towards astrocytes. MHC-class-I was upregulated in reactive astrocytes of all biopsies and two autopsies but not in healthy controls. Importantly, we observed a prominent immunoreactivity of astrocytes with the complement factor C4d. Finally, we provided insight into an early phase of GFAP autoimmunity in an autopsy of a pug dog encephalitis that was characterized by marked meningoencephalitis with selective astrocytic damage with loss of GFAP and AQP4 in the lesions.

Our histopathological findings indicate that a cytotoxic T cell-mediated immune reaction is present in GFAP autoimmunity. Complement C4d deposition on astrocytes could either represent the cause or consequence of astrocytic reactivity. Selective astrocytic damage is prominent in the early phase of GFAP autoimmunity in a canine autopsy case, but mild or absent in subacute and chronic stages in human disease, probably due to the high regeneration potential of astrocytes. The lymphocytic and granulomatous phenotypes might reflect different stages of lesion development or patient-specific modifications of the immune response. Future studies will be necessary to investigate possible implications of pathological subtypes for clinical disease course and therapeutic strategies.

**Supplementary Information:**

The online version contains supplementary material available at 10.1007/s00401-023-02678-7.

## Introduction

Anti-glial fibrillary acidic protein (GFAP) meningoencephalomyelitis (also known as autoimmune GFAP astrocytopathy) is an inflammatory autoimmune disease of the central nervous system (CNS), which is defined by (1) the detection of immunoglobulin type G (IgG) autoantibodies against the alpha (α) GFAP isoform (as well as epsilon [ε] and kappa [κ]) that are diagnostic in the cerebrospinal fluid (CSF) and (2) clinical manifestation of meningitis (fever, headache, neck stiffness, vomiting), encephalitis (altered consciousness, tremor, psychiatric abnormalities, epileptic seizures), myelitis (sensory symptoms and weakness), and/or optic disk edema [10, 12]. Some patients may only display a limited set of symptoms [8, 12, 17, 20, 22, 23]. On brain magnetic resonance imaging (MRI) 23–53% of patients demonstrate linear perivascular radial gadolinium enhancement perpendicular to the ventricles [8, 9, 12, 20, 23, 45, 47]. Around 70% of patients have a monophasic course and readily respond to high dose corticosteroids [8, 12, 17, 23, 49]. Some have concomitant anti-neuronal and anti-glial autoantibodies (11–40%) [8, 9, 12, 25, 50] and in approximately 25% of patients a coexisting neoplasm is detected, most commonly ovarian teratoma [8, 12, 17, 23, 24]. In such a paraneoplastic setting, the ectopic GFAPα expression is considered to trigger an autoimmune response against the physiological astrocytic protein in the CNS. In contrast to antigens that are expressed on the extracellular surface of the target cell and are directly accessible by autoantibodies, GFAPα is an intracellular antigen and the autoantibodies are most likely non-pathogenic but the disease is triggered and propagated by a T cell-mediated immune response [17, 46]. In idiopathic patients the trigger for GFAP autoimmunity remains elusive. In studies performed so far, no anti-GFAP autoantibodies have been detected in a series of patients with multiple sclerosis, traumatic brain injury, or infectious diseases [8, 12]. Autoantibodies against GFAPα were originally described in pug dogs with ataxia, generalized seizures, and depression and presented with three distinct neuropathological phenotypes including granulomatous meningoencephalomyelitis (GME), necrotizing meningoencephalitis (NME), and necrotizing leukoencephalitis (NLE) [26, 30, 38, 42, 43]. In contrast, neuropathological studies in human tissues are rare and limited to a few biopsy studies [11, 17, 19, 23, 39, 52] and one autopsy report [48].

Here we provide an in-depth neuropathological description of anti-GFAP meningoencephalomyelitis based on nine biopsy specimens, two autopsies and one canine autopsy case.

## Materials and methods

### Patient information and inclusion criteria

This study was approved by the Institutional Review Board of Mayo Clinic, Rochester (IRB No. 2067–99), the Ethics Committee of the Medical University of Vienna (EK. No. 1123/2015), and the Institutional Review Board of the University of Schleswig–Holstein (#13–162). Inclusion criteria were: (1) CSF positivity for the specific GFAP-IgG staining by indirect immunofluorescence on murine brain tissue and confirmation by a GFAPα-transfected cell-based assay (CBA) using fixed and permeabilized human embryonic kidney (HEK293T) cells, as previously described [8] without other known detectable coexisting autoantibodies; (2) clinical history available for review; (3) CNS biopsy/autopsy available for pathological analysis. In total nine human biopsy specimens, two human autopsies and one canine autopsy case met the criteria and were included in this study. As controls, we included 16 autopsy brains without GFAPα autoantibodies (tested in CSF): anti-Ma2-associated encephalitis (n = 1), anti-neuronal autoimmune encephalitis (anti-NMDAR n = 3, anti-AMPAR encephalitis n = 2), Alzheimer’s disease (n = 2), MOG antibody associated disorders (n = 1), stroke (n = 1) and healthy controls (n = 6).

### Anti-GFAP autoantibody assessment–immunofluorescence assay (IFA) and fixed cell-based assay (CBA)

All patients’ CSF were tested in the Mayo Clinic Neuroimmunology Laboratory by an indirect immunofluorescence assay (IFA) on murine composite tissue (brain, gut and kidney) and were positive for the specific GFAP-IgG staining as previously described [8, 10].

In brief, cryosections were fixed (4% paraformaldehyde [PFA], 1 min), washed (phosphate-buffered saline [PBS]), permeabilized (0.5% CHAPS [C32H58N2O7S], 1 min), and then washed again (PBS). Normal goat serum (10% diluted in PBS) was applied for 1 h and then sections were incubated with patient’s CSF (1:2 dilution) for 40 min, washed, incubated with secondary antibodies for 30 min (goat anti-human IgG [FITC-conjugated], Southern Biotech, Birmingham, AL), washed and mounted (ProLong Diamond Antifade Mountant, Life Sciences).

In addition, all patients were positive for GFAPα-IgG confirmed by CBA as previously described [8, 10]. Briefly, HEK293T cells stably transfected with a plasmid expressing the human GFAPα isoform, fused with green fluorescence protein (GFP) in individual wells were fixed (4% PFA, 15 min), washed (PBS), permeabilized (0.2% Triton X-100, 10 min) and washed (PBS). Cells were blocked for non-specific secondary antibody binding by incubation for 30 min with 10% normal goat serum. CSF was applied (1:4 dilution) for 1 h, cells were washed and then secondary antibodies (goat anti-human IgG, Southern Biotech, Birmingham, AL or goat anti-dog IgG [both TRITC-conjugated]) were applied for 45 min. Slides were washed and coverslipped using Prolong Antifade Gold mounting media (Life Sciences).

Positivity with both above described assays was necessary for confirmation of GFAP-IgG positivity in the CSF of the included patients.

For the canine autopsy case, an in-house tissue-based assay (TBA) on adult rat brain cryosections was performed at the Division of Neuropathology and Neurochemistry, Department of Neurology, Medical University of Vienna, Austria. Briefly, slides were incubated with 0.3% hydrogen peroxide (H_2_O_2_) for 15 min to block the endogenous peroxidase, washed for three times (PBS) and then blocked again for 1.5 h with 5% normal donkey serum to prevent unspecific protein binding. Then, sections were incubated with canine patient’s CSF (1:2 dilution) over night at 4 °C. Next day, slides were washed for three times (PBS) and the secondary antibody staining was performed automatically on the Autostainer Link 48 system (Dako/Agilent) using the avidin–biotin horseradish peroxidase complex system and the chromogen 3,3′-diaminobenzidine (DAB; Dako/Agilent) resulting in a brown precipitate. The canine GFAPα autoantibodies were confirmed with an in-house CBA as described above (canine anti-GFAPα autoantibodies cross react with the human isoform).

### Magnetic resonance imaging (MRI)

MRI of the two human autopsy cases was performed at the University Clinic St. Pölten using a 3 T MAGNETOM MRI system (Siemens Tim Trio) and at the University of Schleswig–Holstein (3 T Siemens Vida and 1.5 T Siemens Aera). Clinical routine sequences including post-contrast T1 as well as T2-weighted images, fluid attenuated inversion recovery (FLAIR)/Dark fluid turbo inversion recovery magnitude (TIRM) images and susceptibility weighted images (SWI) were acquired. The patients received an intravenous injection of 10–14 ml gadoteric acid (Dotarem®) or gadobutrol (Gadovist®) for acquisition of post-contrast T1-weighted images. The patients were imaged at least twice with the same protocol. MRI of the biopsy patients was done as part of the clinical testing including T2-weighted and FLAIR sequences as well as T1-weighted with gadolinium; all images were reviewed to confirm the compatibility with GFAP autoimmunity and post biopsy imaging was reviewed to confirm the biopsy was done within a lesion.

### Neuropathological evaluation

Neuropathological analysis was performed on brain biopsy material (lesional focus) and on multiple brain regions from the two human autopsy cases including double hemispheric sections and one dog with NME. Formalin-fixed and paraffin-embedded tissue sections were stained with H&E, and with Luxol fast blue-Periodic acid Schiff (LFB/PAS) myelin staining.

### Immunohistochemistry

Immunohistochemistry for the following primary antibodies was performed on the automated platform Autostainer Link 48 using the EnVision™ FLEX + secondary system (Dako/Agilent) according to the manufacturer’s protocol: **alpha-synuclein** (alpha-synuclein oligomers, aa 47–52; mouse clone 5G4; 1:4,000; Analytik Jena), **APP** (amyloid precursor protein; acute axonal damage; mouse clone 22C11; 1:8,000; Millipore), **beta-amyloid** (extracellular beta-amyloid; mouse clone 6F/3D; 1:100; Dako/Agilent), **C4d** (complement split product 4d; rabbit polyclonal; 1:100; Biomedica), **CD3** (T cells; rabbit clone SP7; 1:100; NeoMarkers/Thermo Fisher Scientific), **CD4** (major histocompatibility complex (MHC) class II-restricted T helper cells; mouse clone 4B12; 1:100; Dako/Agilent), **CD8** (MHC class I-restricted cytotoxic T cells; mouse clone C8/144B; 1:100; Dako/Agilent), **CD20** (B cells; mouse clone L26; 1:400; Dako/Agilent), **CD68** (110-kD transmembrane lysosomal glycoprotein in macrophages; mouse clone KP1; 1:5,000; Dako/Agilent), **CD79a** (lymphoblasts and plasma cells; mouse clone JCB117; 1:100; Dako/Agilent), **CD138** (plasma cells; mouse clone B-A38; 1:25; Cell marque), **DLA** (dog leukocyte antigen; mouse clone TAL.1B5; 1:1,000; Dako/Agilent), **GFAP** (glial fibrillary acidic protein; astrocytes; rabbit polyclonal; 1:6,000; Dako/Agilent), **HLA-DR** (MHC class II antigen; mouse clone CR3/43; 1:400; Dako/Agilent), **Iba-1** (Ionized calcium-binding adapter molecule 1; rabbit polyclonal; 1:3,000; Wako), **IgG** (immunoglobulin type G heavy chains; plasma cells, rabbit polyclonal; 1:16,000; Dako/Agilent), **Ki-67** (proliferation; mouse clone MIB-1; 1:200; Dako/Agilent), **perforin** (pore forming cytolytic protein; mouse clone 5B10; 1:50; Leica)**, pSTAT1** (interferon signaling; rabbit clone 58D6; 1:200; Cell Signaling Technology), **pTDP-43** (transactive response DNA-binding protein 43, phospho-Ser409/410; mouse clone 11–9; 1:20,000; Cosmo Bio), and **Tau** (phosphatase sensitive epitope on PHF-Tau phospho-Ser202/Thr205; mouse clone AT8; 1:200; Thermo Fisher Scientific). Heat-induced epitope retrieval (HIER) was performed either with target-retrieval solution low pH (Dako/Agilent) for alpha-synuclein, APP, CD8, CD20, CD68, CD79a, DLA, HLA-DR, Iba-1, IgG, p62, perforin and pTDP-43 or with target-retrieval solution high pH (Dako/Agilent) for C4d, CD3, CD4, CD138, Ki-67, and pSTAT1. Concentrated formic acid pretreatment for 1 min was used for alpha-synuclein and pTDP-43 in addition to HIER with low pH. For beta-amyloid staining pretreatment with 80% formic acid (aqueous solution) for 1 h was used. Pretreatment with proteinase K (5 min; Dako/Agilent) was used for the GFAP staining. Staining for Tau was done in sections without pretreatment.

Manual immunohistochemistry was performed in a humidified chamber for **AQP1** (aquaporin 1; water channel; rabbit polyclonal; 1:500; Santa Cruz), **AQP4** (aquaporin 4; astrocytic water channel; rabbit polyclonal; 1:250; Sigma Aldrich), **C1q** (complement-mediated tissue injury; mouse clone [C1QA/2956]; 1:1,000; Abcam), **C9neo** (complement C9 neoantigen; complement-mediated tissue injury; rabbit polyclonal; 1:2,000; from Professor Paul Morgan, Cardiff, UK), **CD103** (integrin protein encoded by the *ITGAE* gene; rabbit clone EPR4166(2); 1:2,000; Abcam), **cleaved caspase 3** (apoptosis; Asp175; rabbit clone 5A1; 1:100; Cell Signaling), **granzyme A** (cell lysis in cell-mediated immune responses; rabbit clone EPR20161; 1:100; Abcam), **granzyme B** (cell lysis in cell-mediated immune responses; mouse clone GZB01; 1:1,000; LabVision/Thermo Fisher Scientific), **MHC class I** (major histocompatibility complex class I; for autopsies: mouse clone HC10; 1:4000; gift from Hidde Ploegh [40]; for biopsies: mouse monoclonal; 1:200; Santa Cruz) and **Neuropilin 1** (cellular complement receptor; rabbit clone EPR3113; 1:100; Abcam) using the avidin–biotin-complex method. No pretreatment was necessary for AQP1, and AQP4. For the C9neo staining enzymatic pretreatment with proteinase type 24 was used. HIER with ethylenediaminetetraacetic acid (EDTA) buffer pH 9.0 was used for the C1q, granzyme A, granzyme B and the MHC class I staining, while HIER with low pH (pH 6.0) was used for CD103, cleaved caspase 3 and Neuropilin 1. For the detection of DNA fragmentation, terminal deoxynucleotidyl transferase dUTP nick end labelling (**TUNEL)** was performed using the In Situ Cell Death detection kit (alkaline phosphatase; Roche). Double labelling of different antigens was performed through the chromogenic reactions with the two different substrates Fast Blue [blue] and 3-amino-9-ethylcarbazole (AEC; Dako/Agilent) [red].

Image acquisition was performed using a NanoZoomer 2.0-HT digital slide scanner C9600 (Hamamatsu Photonics, Hamamatsu, Japan) and Olympus BX51 microscope (EVIDENT, Tokyo).

### Immunofluorescence

For immunofluorescence double labeling with GFAP and Ki-67, tissue sections were first deparaffinized and blocked with endogenous peroxidase against non-specific binding for at least 10 min. Afterwards, HIER with high pH (pH 9.0) was performed and the slides were incubated overnight at 4 °C with a mixture of the primary antibodies of different species against GFAP and Ki-67. The next day, slides were incubated with fluorescently labeled secondary antibodies [anti-rabbit Cy3 for GFAP (goat polyclonal; 1:1000; Jackson ImmunoResearch) and anti-mouse Alexa Fluor 488 for Ki-67 (goat polyclonal; 1:800; Jackson ImmunoResearch)] for 2 h at room temperature. Finally, nuclei were visualized with 4′,6-diamidino-2-phenylindole (DAPI; 1 µg/ml; Invitrogen) for 5 min at room temperature and mounted with Aqua-Poly/Mount (PolySciences). Slides were dried overnight at 4 °C in the dark and analyzed the next day with a confocal laser microscope LSM700 (Zeiss, Oberkochen, Germany).

### Quantitative analysis

The slides were reviewed with respect to the presence, extent, and distribution of inflammation, inflammatory cell types, axonal damage, astrocyte damage, and demyelination. To investigate a putative cytotoxic T cell-mediated attack against astrocytes, we evaluated MHC class I expression on the astrocytes and granzyme A, B and perforin in T lymphocytes. A topographic relationship was analyzed by double labeling of cytotoxic T cells markers and GFAP.

The topographical distribution of B cells in anti-GFAP meningoencephalomyelitis was assessed by staining a double hemispheric section (autopsy case #1) with an anti-CD20 antibody. The stained section was scanned and immunolabelled cells were mapped as red dots using the bioimage analysis platform QuPath-0.2.2 [2].

## Results

The neuropathological analysis of human autopsy and biopsy cases of a total of 11 patients with anti-GFAP meningoencephalomyelitis revealed two distinct inflammatory phenotypes, a lymphocytic and a granulomatous phenotype.

### Autopsy cases–clinical, neuropathological, and neuroimaging features

### Autopsy Case #1, Lymphocytic phenotype

A 75-year-old woman presented with visual and auditory hallucinations and delusive thinking. Six months later she developed fever (39 °C), disorientation and headache and was admitted to the hospital. Initial CSF analysis revealed 403 white blood cells (WBCs) (normal, 0–5) with predominant lymphocytes, plasma cells and some eosinophilic granulocytes, total protein of 1325 mg/L, and CSF-specific (serum unmatched) oligoclonal bands. EEG showed a diffuse slowing and epileptic discharges left frontal and temporal. Extensive infectious and neoplastic workup was negative. The patient received ceftriaxone, ampicillin/sulbactam and acyclovir as well as levetiracetam. Despite treatment the symptoms of the patient worsened, and she developed dysarthria, rigor of the upper extremities and ataxia. Follow-up lumbar puncture continued to show elevated cell count (122 WBCs – mainly lymphocytes and plasma cells). Because the patient did not respond to antibiotic treatment, an autoimmune encephalitis was considered, and high-dose steroids were administered. MRI two weeks before death revealed mild brain atrophy with dilated lateral ventricles and bilateral hyperintensities in the periventricular gray and white matter and left capsula externa. Despite steroids, the patient deteriorated and died of cardio-respiratory failure eight months after disease onset. GFAPα-IgG was confirmed post-mortem.

**Fig. 1:**
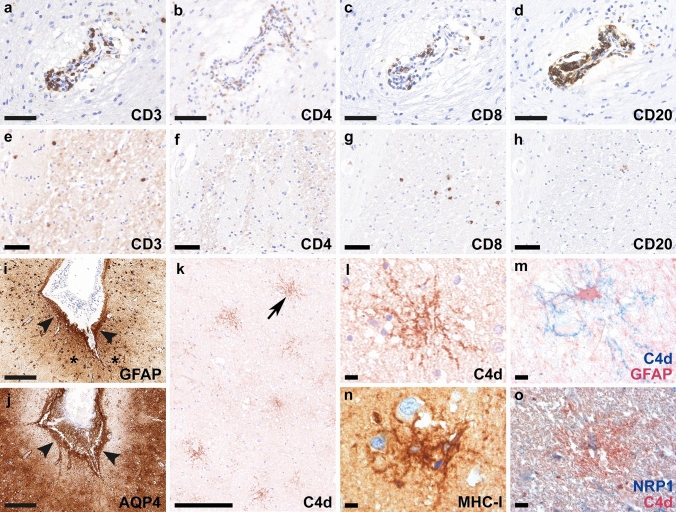
Neuropathological findings of anti-GFAP meningoencephalitis – autopsy case #1 The inflammatory reaction is characterized by perivascular cuffs with abundant CD3^+^ **(a)**, CD4^+^ **(b)** and CD8^+^ T cells **(c)**, as well as CD20^+^ B cells **(d)**. In the brain parenchyma, CD3^+^ **(e)** and CD8^+^ T cells **(g)** were more abundant compared to CD4^+^ **(f)** and CD20^+^ **(h)** lymphocytic infiltrates. Reactive gliosis in the depth of the sulci (**i**, asterisks, GFAP) is present subjacent to meningeal lymphocytic infiltrates (**i**, arrowheads, GFAP). AQP4 is upregulated and mainly found in subpial astrocytes (**j**, arrowheads). C4d indicates complement deposition on astrocytes **(k,** arrow in** k** enlarged in panel **l)**, confirmed with double labeling for C4d (blue) and GFAP (red) **(m)**. MHC class I molecules are upregulated in some astrocytes **(n)**. Deposition of C4d on NRP1-positive glial cells is confirmed with the double labeling for NRP1 (blue) and C4d (red) **(o)**. Scale bars **a–d, i, j** 100 µm; **e–h** 50 µm; **k** 500 µm; **l-o** 25 µm. *AQP4* aquaporin 4, *CD* cluster of differentiation, *C4d *complement split product 4d, *GFAP* glial fibrillary acidic protein, *MHC-I* major histocompatibility complex class I, *NRP1* neuropilin 1

Post-mortem examination of the entire brain revealed a lymphocytic meningoencephalitis characterized by abundant perivascular lymphocytic infiltrates involving the gray and white matter throughout all brain regions with a focus on basal ganglia and brainstem. Lymphocytic infiltrates (Fig. 1a–d) mainly consisted of CD3^+^ (Fig. 1a), CD4^+^ (Fig. 1b) and CD8^+^ T cells (Fig. 1c) and CD20^+^ B cells (Fig. 1d); CD3^+^/CD8^+^ T cells were more abundant in the brain parenchyma compared to CD4^+^ T cells and CD20^+^ B cells (Fig. 1e–h). In addition, a moderate number of parenchymal and perivascular CD79a^+^ B cells/plasma cells were observed, while only a few CD138^+^ plasma cells were present in the brain parenchyma and around the vessels (data not shown). H&E staining revealed few eosinophilic granulocytes in the perivascular region (data not shown). The CNS tissue showed widespread reactive gliosis with prominent accentuation of activated astrocytes, and microglia and macrophages with upregulated HLA-DR around inflamed vessels. Moreover, the astrocytes showed a prominent band-like subpial reactive gliosis in topographical association with meningeal inflammation in the depth of the sulci (Fig. 1i, asterisks and arrowheads, GFAP). AQP4 was upregulated and mainly found in subpial astrocytes (Fig. 1j, arrowheads). Immunohistochemistry for the activated complement split product C4d revealed a strong labeling along cell membranes and processes of a substantial number of astrocytes throughout the gray and white matter including cerebral cortex, hippocampus, amygdala, hypothalamus, basal ganglia, subcortical and deep white matter, brainstem and cerebellar white matter (Fig. 1k, arrow in k enlarged in l; m). The C4d-labeled astrocytes did not express apoptotic markers such as cleaved caspase 3 or TUNEL, however, some astrocytes showed a strong upregulation of MHC class I (Fig. 1n). In addition, upregulation of neuropilin 1 (NRP1; CD304), a transmembrane glycoprotein that has been shown to serve as cellular complement receptor [4], was found on glial cells, some of them with co-expression of C4d on the membrane (Fig. 1o). Some perivascular and parenchymal T cells in proximity to astrocytes were granzyme A positive (see autopsy case #2 and biopsy findings below). No acute axonal damage with accumulation of APP was present in association with inflammatory infiltrates. We did not detect deposition of IgG, C1q, and terminal complement complex C9neo on astrocytes.

In addition, the patient revealed a pronounced Alzheimer’s disease pathology with numerous neurofibrillary tangles, dystrophic neurites and neuritic plaques (Braak & Braak neurofibrillary stage V; CERAD neuritic plaque score C; Thal amyloid phase 4; NIA/AA criteria: A3, B3, C3). The amyloid in the neuritic plaques was also labeled with the complement components C1q and C4d, but astrocytes did not show a specific C1q labeling. Additionally, the patient showed prominent amyloid angiopathy. Alpha synuclein pathology or pTDP43 inclusion bodies were not detected (data not shown). Two additional Alzheimer’s disease cases without anti-GFAP autoantibodies in CSF (used as a “control”) showed complement C4d deposition only in neuritic plaques but not on astrocytes.

**Fig. 2:**
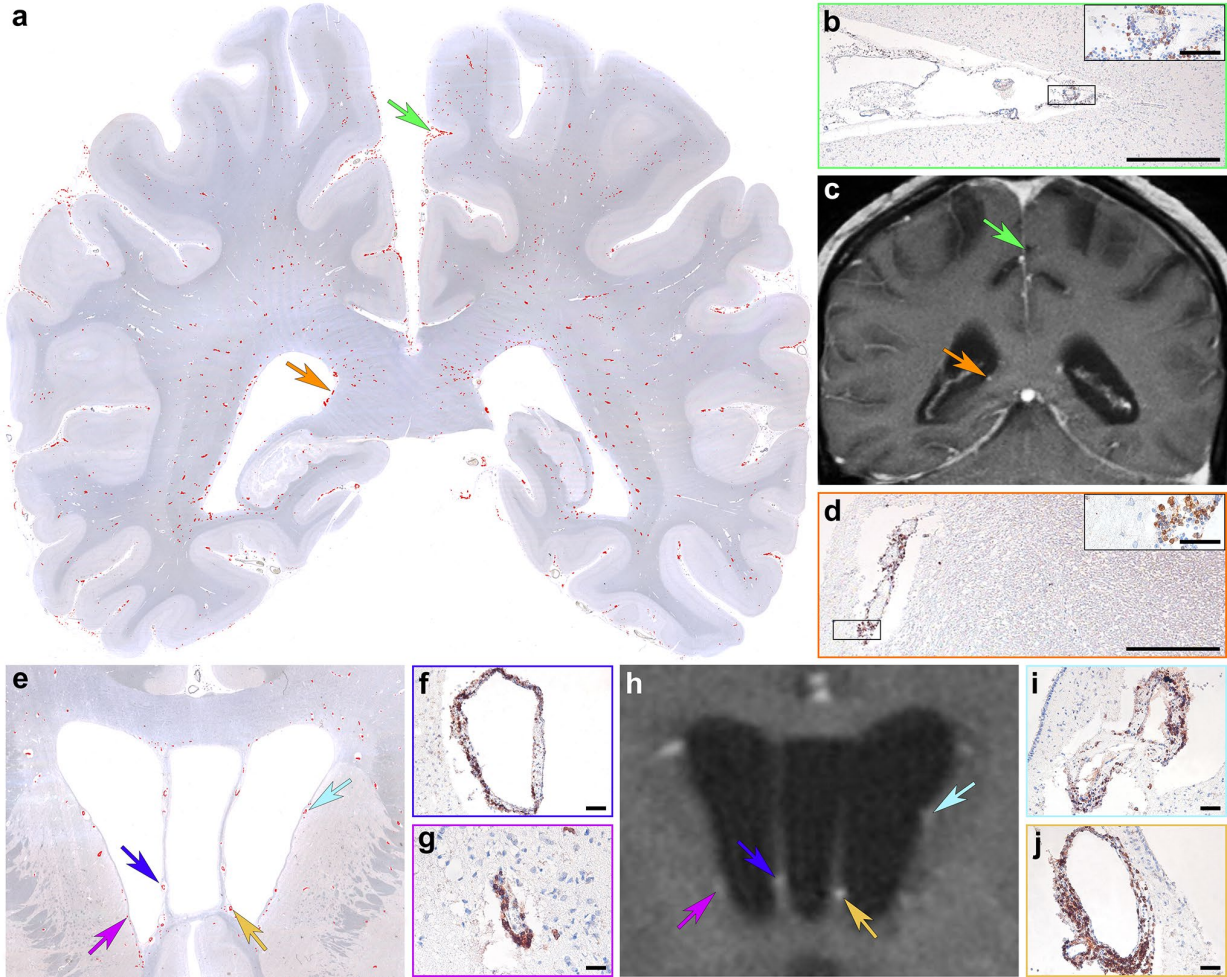
Topographical mapping and imaging characteristics of inflammatory infiltrates of autopsy case #1 Topographical evaluation of a double-hemispheric brain section shows a widespread distribution of CD20^+^ B cells (**a**; red dots represent immunolabeled cells) in the meninges (**a**, green arrow enlarged in panel **b**), in perivascular cuffs (**a**, orange arrow enlarged in panel **d**) and in the brain parenchyma. Corresponding in vivo brain MRI shows multiple areas of gadolinium enhancement in post-contrast T1 images **(c,** green arrow indicates meningeal inflammation, orange arrow indicates the corresponding place where abundant number of B lymphocytes were present around blood vessels). Anatomic distribution of inflammation in a more anterior brain section including the basal ganglia similarly shows a maximum of CD20^+^ B cells mainly around the ventricles (**e**; red dots represent immunolabeled cells). Colored arrows in **e** and** h** (enlarged in **f**, **g**, **i**, **j**) indicate a profound number of perivascular CD20^+^ B cells that are visible as gadolinium enhancement in the corresponding in vivo MR image **(h)**. Scale bars **b**, **d** 500 µm; **inset** in** b** and** d** 50 µm; **f**, **g**, **i**,** j** 25 µm

Topographic mapping of CD20^+^ B cells in a double hemispheric brain section revealed a widespread distribution with a predominance in the leptomeninges and in perivascular cuffs in the deep white matter (Fig. 2a, b). In vivo 3 Tesla post-contrast T1 MR images showed multiple areas of perivascular gadolinium enhancement in the white matter perpendicular to the ventricles (Fig. 2c) that neuropathologically corresponded to dilated Virchow–Robin spaces with abundant CD20^+^ lymphocytic infiltrates (Fig. 2d–j).

### Autopsy Case #2, Granulomatous phenotype

**Fig. 3:**
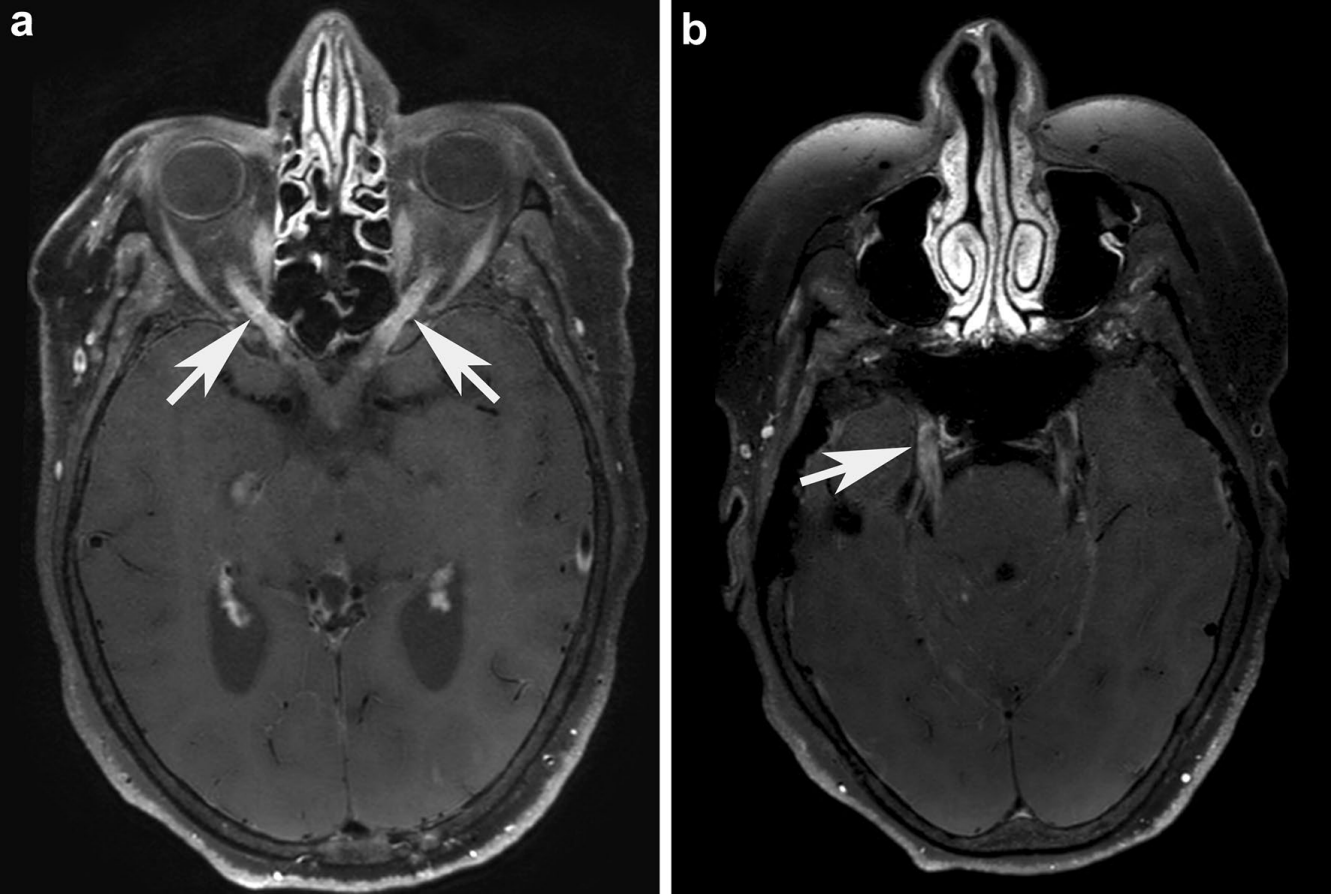
Magnetic resonance imaging findings of anti-GFAP meningoencephalomyelitis – autopsy case #2 Brain in vivo MRI from autopsy patient #2 shows a bilateral optic neuritis (**a**, white arrows) on post-contrast axial angulated reconstructions of T1-weighted blackblood-images. Further axial angulated reconstruction of post-contrast T1-weighted blackblood-images shows enhancement throughout the cisternal segment of both trigeminal nerves **(b)**. The right trigeminal nerve additionally shows an abnormal enhancement in the trigeminal cave (**b,** white arrow), indicating an involvement of the ganglion

A 54-year-old woman presented with subacute-chronic progressive weakness of the legs, back pain and vertigo followed by behavioral changes, disorientation, aphasia, bilateral visual and hearing loss. On examination, she had severe paraparesis and sensory ataxia of the legs, reduced vibration sensation of the ankles, severe visual loss with bilateral (left >  > right) optic disc swelling, hearing loss, severe aphasia and agitation. CSF showed 77 WBC (mainly lymphocytes), elevated total protein 1838 mg/L, increased lactate 5.92 mmol/L (< 2.5) and reduced glucose 1.79 mmol/L (2.8–4.2, CSF/serum glucose 0.27, normal > 0.5). Soluble interleukin 2 (IL2) receptor was elevated in CSF 356 kU/L (< 50). Infectious work-up for viruses, bacteria including tuberculosis and lues, toxoplasmosis and fungi was negative. Clinical work-up for sarcoidosis was negative. During the course of the disease, four brain MRIs were performed and showed prominent basal and parietooccipital gadolinium enhancement, bilateral anterior optic neuritis (Fig. 3a, white arrows) as well as bilateral trigeminal nerve enhancement (Fig. 3b). In addition, asymmetric cerebellar T2-hyperintensities with localized paramagnetic pigment or “microbleeds” were detected. In the spinal cord, diffuse dorsal column- predominant multi-level myelitis was found. Extensive infectious and meningeal neoplasia workup was negative. The patient received ceftriaxone, ampicillin/sulbactam and acyclovir but she did not respond. After detection of serum and CSF anti-GFAP autoantibodies, she was treated with intravenous methylprednisolone (5 × 1000 mg) followed by oral tapering over 8 weeks. She rapidly responded, and vision, gait, disorientation, and MRI changes improved. She was electively readmitted one month later, still on 50 mg prednisolone/day, and her MRI showed further improvement, but she was found dead the following day due to fulminant pulmonary embolism based on deep vein thrombosis in spite of prophylactic antithrombotic therapy.

**Fig. 4:**
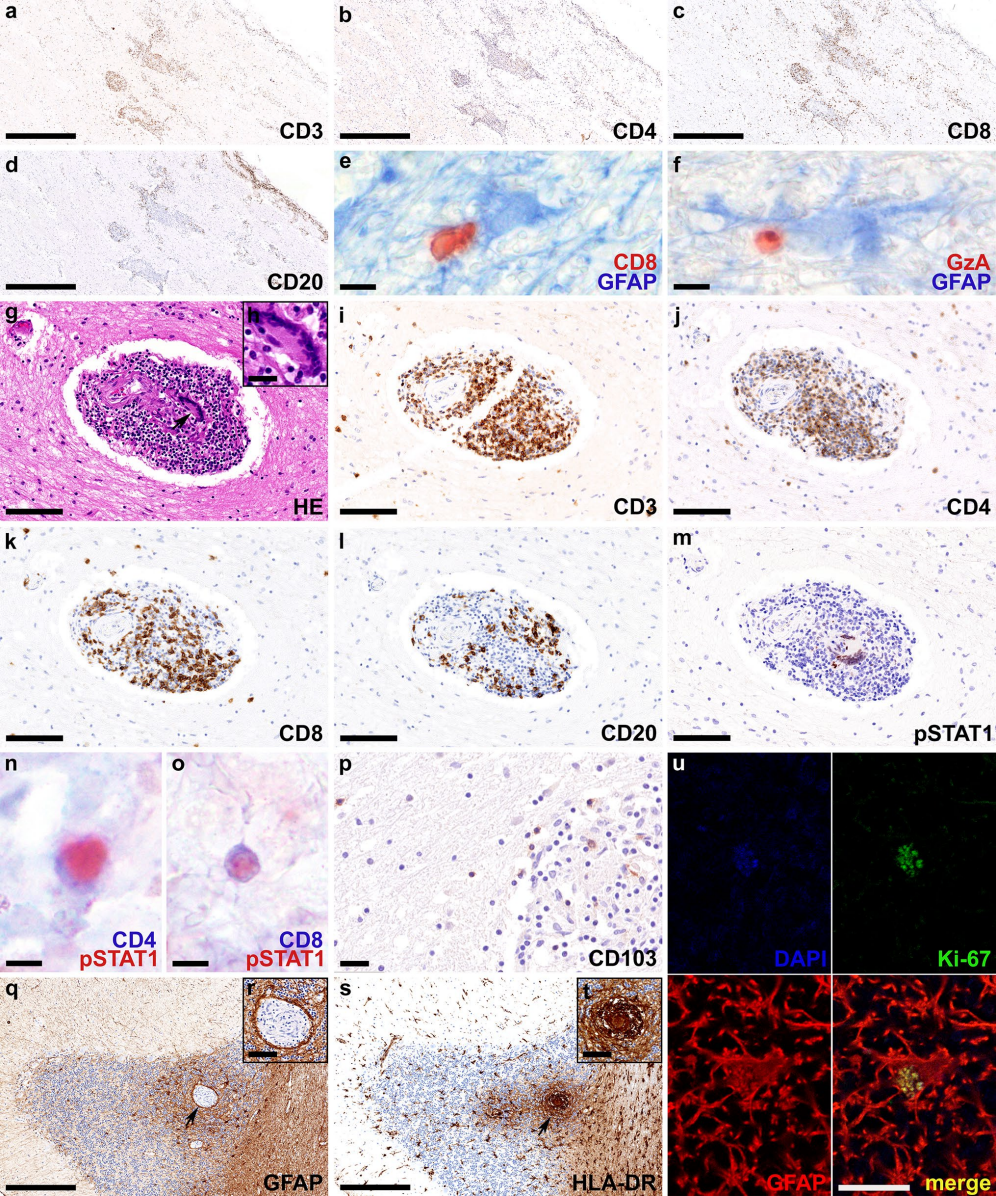
Neuropathological findings of anti-GFAP meningoencephalomyelitis – autopsy case #2 In the chiasma opticum numerous CD3^+^ **(a)**, CD4^+^ **(b)** and CD8^+^ T cells **(c)** and CD20^+^ B cells **(d)** are present in the leptomeninges and around vessels. Parenchymal CD8^+^ T cells (red) are found in close proximity to GFAP positive astrocytes (blue) **(e)**. Some of these T cells express granzyme A (red) **(f)**. The perivascular granulomas contain multinucleated giant cells of the Langhans type (arrow in** g** enlarged in inset **h**), and a high number of CD3^+^ **(i)**, CD4^+^ **(j)** and CD8^+^ T cells **(k)**, and less CD20^+^ B cells **(l)**. pSTAT1, a marker for interferon signaling, is strongly upregulated in nuclei of multinucleated giant cells **(m)** and CD4^+^ **(n)** and CD8^+^ T lymphocytes **(o)**. CD103^+^ tissue resident memory T cells are present in the granulomas as well as in the brain parenchyma **(p)**. In the cerebellum, granulomas are evident in the cortex with prominent astrogliosis (arrow in **q** enlarged in inset **r**) with well-preserved astrocytic processes **(r)** and microglia activation (arrow in **s** enlarged in inset **t**). The proliferation marker Ki-67 (green) is expressed in the nucleus (blue; DAPI) of some astrocytes (red; GFAP) **(u)**. Scale bars **a-d, q, s** 250 µm; **e, f, n, o** 6 µm; **g, i-m** 50 µm; **h, p, r, t** 10 µm; **u** = 5 µm. *AQP4* aquaporin 4, *CD* cluster of differentiation, *DAPI* 4’,6-diamidino-2-phenylindole, *GFAP* glial fibrillary acidic protein, *GzA* granzyme A, *H&E* hematoxylin & eosin, *HLA* human leukocyte antigen, *pSTAT1* phosphorylated signal transducer and activator of transcription 1

**Fig. 5:**
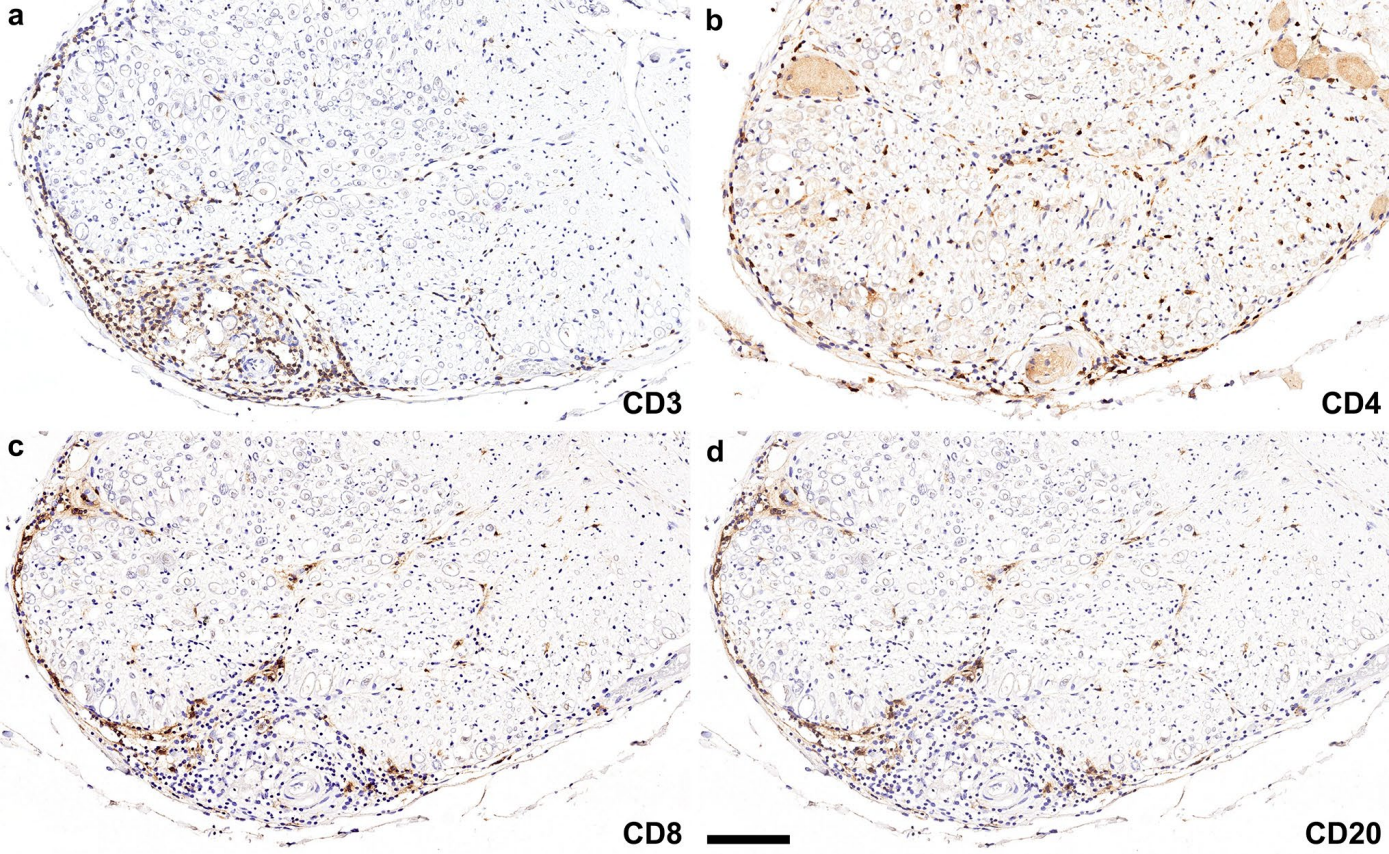
Inflammatory infiltrates in the posterior spinal roots of autopsy case #2 In the spinal roots, CD3^+^ **(a)**, CD4^+^ **(b)**, CD8^+^ **(c)**, and CD20^+^ **(d)** lymphocytes are predominantly found in the peri- and epineurium, in addition scattered lymphocytes are found in the endoneurium. Scale bars **a-d** 100 µm. *CD* cluster of differentiation

Post-mortem brain tissue samples were obtained from the neocortex, basal ganglia, thalamus, hypothalamus, amygdala, hippocampus, cerebellum, pons, medulla oblongata, optic chiasm, and the cervical, thoracic, and lumbar spinal cord, and showed a granulomatous meningoencephalomyelitis with an angiitic component. Inflammation correlated topographically with the MRI abnormalities and was characterized by CD3^+^, CD4^+^, CD8^+^, and CD20^+^ (Fig. 4a-d) lymphocytic infiltrates that were found predominantly in the leptomeninges and perivascular space. The CD4^+^/CD8^+^ T cell ratio did not significantly differ between leptomeninges (ratio CD4/CD8 temporal lobe: 1.56) and perivascular spaces (ratio CD4/CD8 amygdala: 1.43; hippocampus: 1.19), moreover no ectopic lymph follicles were found. The intraparenchymal inflammatory infiltrates mainly consisted of CD3^+^, CD4^+^ and CD8^+^ T lymphocytes, while CD20^+^ B cells were found predominantly in the perivascular and leptomeningeal compartment. Some parenchymal T cells were found in proximity to astrocytes and were granzyme A positive (Fig. 4e, f). Eosinophilic granulocytes were not visible. Parenchymal inflammation was not accompanied by AQP4 loss (data not shown). The perivascular and leptomeningeal space contained prominent and widespread epithelioid granulomas with multinucleated giant cells (arrow in Fig. 4g enlarged in inset h) in the optic chiasm (Fig. 4g–m), gray and white matter of diencephalon, mesencephalon, cerebellum (Fig. 4n–t), pons, and spinal cord that focally affected the vessel walls and was accompanied by hemosiderin deposition (angiitic component). The granulomas were composed of abundant CD3^+^ (Fig. 4i), CD4^+^ (Fig. 4j) and CD8^+^ (Fig. 4k) T cells but less CD20^+^ B cells (Fig. 4l) and showed a focal pSTAT1 expression in the nuclei of multinucleated giant cells (Fig. 4m) and CD4^+^ and CD8^+^ T cells (Fig. 4n, o). In addition, we found abundant CD103^+^ tissue resident memory T cells (Fig. 4p). Quantification of CD103^+^ cells in the cerebellum and pons revealed 26 cells/mm^2^, which was more abundant compared to the lymphocytic autopsy case #1 (8 cells/mm^2^). Astrocytes were well preserved and showed reactive proliferation (reactive astrogliosis) combined with prominent microglial activation with upregulation of HLA-DR around inflamed vessels and granulomas (Fig. 4q–t). Some of the astrocytes revealed a nuclear expression of the proliferation marker Ki-67 (Fig. 4u). We did not detect deposition of IgG, C1q, C4d, or terminal complement complex C9neo on astrocytes. CD3^+^, CD4^+^, CD8^+^, and CD20^+^ lymphocytes were also present in the epi- and perineurium of the anterior and posterior spinal nerve roots, in addition scattered lymphocytes were present in the endoneurium (Fig. 5a–d). We did not detect GFAP immunoreactivity in the Schwann cells or fibroblasts (data not shown). Post-mortem samples of lung, heart, liver, spleen, pancreas, thyroid gland, kidney, suprarenal gland, and bone marrow did not reveal any granulomas.

### Biopsy cases–inflammatory and neuropathological features

The biopsies were all obtained from brain and the overall male/female ratio was 5:4. The median pre-biopsy duration of symptoms was 8 (3–13) months. Two patients received steroid treatment before biopsy. None of the patients had an underlying neoplasm. Detailed clinical information is listed in Table 1.

**Table 1:** Clinical and demographic information of the study cohort

Species/Sample types	Sex/Age [years]	Clinical presentation	Prior immuno suppression	Time to biopsy/death (autopsy) [months]	Site of brain biopsy/autopsy	Treatment	Disease duration/Overall outcome	Neuropathological phenotype
Canine/Autopsy	F/5	NME, seizures, ataxia	No	3 days	Brain autopsy	Antiepileptic	3 days/Death	Acute necrotizing (severe; GM, WM)
Human/Biopsy	M/37	MEM, ataxia^a^	No	3 (current episode)	Right temporal	Steroids, MMF	7 years (current episode)/Normal	Lymphocytic (severe; GM, WM, Figure 7j, o)
Human/Biopsy	F/58	MEM, tremor, ataxia	No	3	Right frontal	Steroids, MTX, IVIg, MMF	10 months/Initial improvement, remaining behavioral and walking difficulties (mRS4); long-term follow-up N/A	Lymphocytic (mild; GM, WM)
Human/Biopsy	M/53	MEM, tremor	No	4	Right frontal	Steroids, MMF	9 years/Improved but persistent tremor (mRS2)	Lymphocytic (moderate; GM, WM, Fig. 8a–o)
Human/Biopsy	F/29	MEM, optic disk edema, tremor	No	5	Right frontal	Steroids, MMF	2 years/Initially improved; after 2nd relapse persistent vertigo and hearing loss (mRS2)	Mixed granulomatous-like/lymphocytic (mild; GM, WM, Fig. 6a–d, u–x**, **Fig. 7d–i)
Human/Biopsy	F/42	Tremor, encephalitis, seizures	No	5	Left temporal & meninges	Steroids, MMF	11 years/Normal	Lymphocytic (moderate; GM, WM)
Human/Autopsy	F/54	MEM, vertigo, behavioral changes, disorientation, hearing loss, aphasia, paraparesis, pallhypesthesia, visual loss, optic disc edema	Yes; steroids	6	Brain and spinal cord autopsy	Steroids	6 months/Death	Granulomatous (severe; GM, WM)
Human/Autopsy	F/75	ME, hallucinations, dysarthria, rigor, tetra-ataxia	Yes; steroids	8	Brain autopsy	Steroids	8 months/Death	Lymphocytic (moderate; GM, WM)
Human/Biopsy	M/51	MEM, tremor	No	8	Right frontal	Steroids, MMF	9 years/Initial improvement; persistent myelopathy (mRS4)	Lymphocytic (moderate; GM, WM, Fig. 6i–t, Fig. 7m)
Human/Biopsy	M/36	MEM with optic disk edema	Yes; steroids	9	Right frontal	Steroids, AZA	11 years/Normal	Lymphocytic (severe; GM, WM, Fig. 6h; Fig. 7b, c)
Human/Biopsy	F/58	MEM, tremor, optic disk edema	Yes; steroids, IFNβ	9	Left frontal	Steroids, MMF, changed for AZA after relapse	3 years/Some tandem gait unsteadiness (mRS1)	Lymphocytic (moderate; WM, Fig. 7k, l, n)
Human/Biopsy	M/49	Cognitive decline, headache, ambulation difficulties^b^	No	13	Right temporal	Steroids, RTX	N/A	Lymphocytic (moderate; GM, WM, Fig. 6e–g)

No co-existing anti-neuronal and anti-glial autoantibodies were detected

*AZA* azathioprine, *F* female, *Fig.* figure, *GM* gray matter, *IFNβ* interferon β, *IVIg* intravenous immunoglobulin, *M* male, *ME* meningoencephalitis, *MEM* meningoencephalomyelitis, *MMF* mycophenolate mofetil, *mRS* modified Rankin Score, *MTX* methotrexate, *N/A* not available, *NME* necrotizing meningoencephalitis, *RTX* Rituximab, *WM* white matter

^a^1^st^ episode with similar presentation 6 years prior that spontaneously improved

^b^The patient presented with severe leukoencephalopathy in the MRI without gadolinium enhancement

**Fig. 6:**
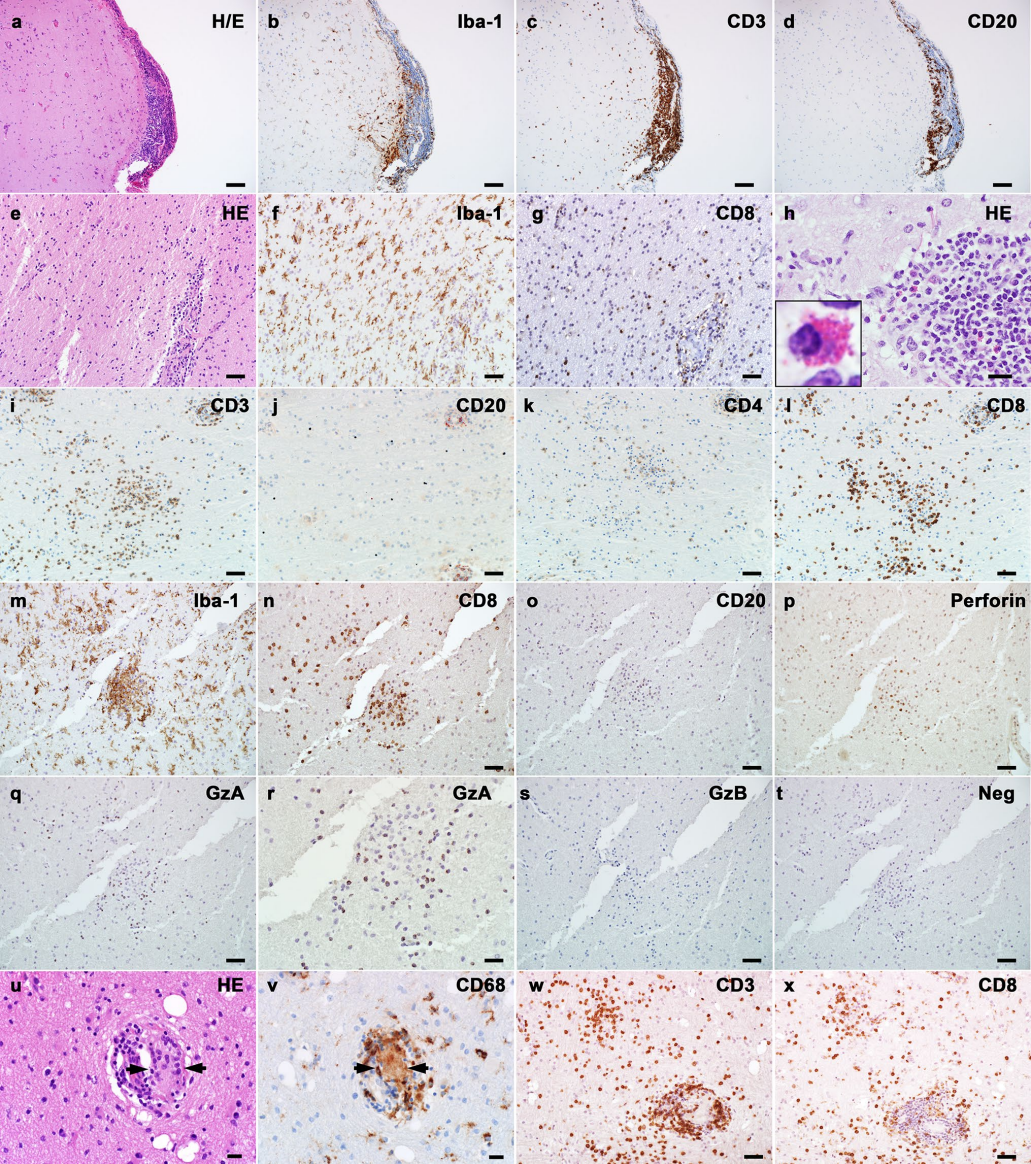
Inflammation involving both CNS gray and white matter in GFAP autoimmunity (a-d; meninges and cortex, biopsy findings) Consecutive sections showing meningeal and gray matter inflammation in the brain: **(a)** H&E stain reveals hypercellularity in both meninges and subpial cortex. **(b)** Marked microglial activation. **(c)** T cell infiltration in both meninges and cortex. **(d)** B lymphocytes remain within the meninges, without cortical parenchymal infiltration. **(e–g**; white matter) Consecutive sections show hypercellularity **(e, H&E)**, microglial activation **(f)** and CD8^+^ T cell **(g)** parenchymal infiltration in the white matter of the brain. **(h, inset enlarged)** Eosinophilic granulocyte infiltration is occasionally seen in the perivascular region. **(i-l;** parenchyma**)** Consecutive sections show diffuse and perivascular inflammation in the brain parenchyma: **(i)** T lymphocytes are present in both parenchyma and perivascular region. **(j)** B lymphocytes are limited to the perivascular space. **(k)** A minority of T lymphocytes are positive for CD4. **(l)** Most T lymphocytes are CD8 positive. **(m-t;** microglial nodules**)** Consecutive sections showing microglial nodules: **(m)** Iba-1 stain indicates microglial nodule formation. **(n)** CD8-positive T lymphocytes are present in the nodule. **(o)** No B lymphocytes are present within the nodule. **(p)** Perforin labels some T lymphocytes. **(q)** Granzyme A (GzA) positive stain indicates cytotoxic T cells, magnified in **(r)**. **(s)** Granzyme B (GzB)-positive cells are absent within this microglial nodule. **(t)** The negative control by omitting the primary antibody indicates the specificity of the immunohistochemistry system. Single biopsy shows mixed lymphocytic and granulomatous patterns of inflammation **(u-x)**: **(u)** One biopsy harbors a multinucleated giant cell (indicated with arrows) present in the perivascular regions with lymphocytic infiltration (H&E). **(v)** Immunohistochemistry on consecutive section reveals the expression of CD68, a phagocytosis marker of myeloid cells, in the multinucleated giant cells (pointed with arrows). **(w)** A different location in the same biopsy shows perivascular and diffuse T cell infiltration (CD3). **(x)** A major proportion of these T cells shows CD8 positivity. Scale bars **a-d** 100 µm; **e–g, i-t, w, × **50 µm; **h, u, v** 20 µm. *CD* cluster of differentiation, *GzA* granzyme A, *GzB* granzyme B, *H&E* hematoxylin & eosin, *Iba-1* ionized calcium-binding adapter molecule 1, *Neg* negative control

In eight out of nine biopsies histopathological findings were consistent with a lymphocytic meningoencephalitis (Fig. 6a–t). One biopsy showed a mixed granulomatous-like/lymphocytic meningoencephalitis with cytotoxic T cells coexistent with perivascular multinucleated giant cells but no mature granuloma formation (Fig. 6u–x).

### Inflammatory involvement of the meninges, cortex, and white matter

Of the nine biopsies, five cases had meninges available for analysis and all showed meningeal inflammatory infiltration. In all cases cortical and white matter inflammation (either or both) were present. Specifically, eight biopsy cases had cortex available, with 7/8 cases positive for cortical inflammation. All biopsies with white matter available demonstrated white matter parenchymal inflammation.

Inflammatory infiltration consisted mainly of T lymphocytes and reactive microglia/macrophages, with fewer B lymphocytes, occasional neutrophils, and eosinophils. T lymphocytes were the dominant component infiltrating the CNS parenchyma and meninges, with variable B lymphocytes mainly restricted to the Virchow-Robin spaces. The marked microglial reactions with scattered microglial nodules were present in both, cortex and white matter (Fig. 6m). Numerous lymphocytes were also present within the microglial nodules. Inflammatory distribution was classified into three patterns: (1) focal perivascular (8/9), characterized by inflammatory cells accumulated in the perivascular space; (2) diffuse parenchymal (7/9); (3) focal microglial nodules or mixed microglial-lymphocytic nodules (7/9).

Seven cases were analyzed for CD4/CD8 ratio and six of them showed CD8 predominance. In the inflamed area (including gray and white matter), CD4^+^ cell density ranged from 0 to 1140 cells/mm^2^, with a median of 66 cells/mm^2^, while CD8^+^ cell density was 44- 822 cells/mm^2^, with a median of 203 cells/mm^2^. We found no correlation between the CD4/CD8 ratio and disease duration. Complement C4d and C9neo stainings were performed in four biopsy cases. Two of four cases showed specific C4d staining along astrocytic membranes and processes like in autopsy case #1, but none showed C9neo immunoreactivity on astrocytes. The CNS biopsies did not show severe axonal damage, except for occasional axonal spheroids. No primary demyelination was observed.

### Cytotoxic T lymphocytes-mediated immune reactions are present in GFAP autoimmunity

**Fig. 7:**
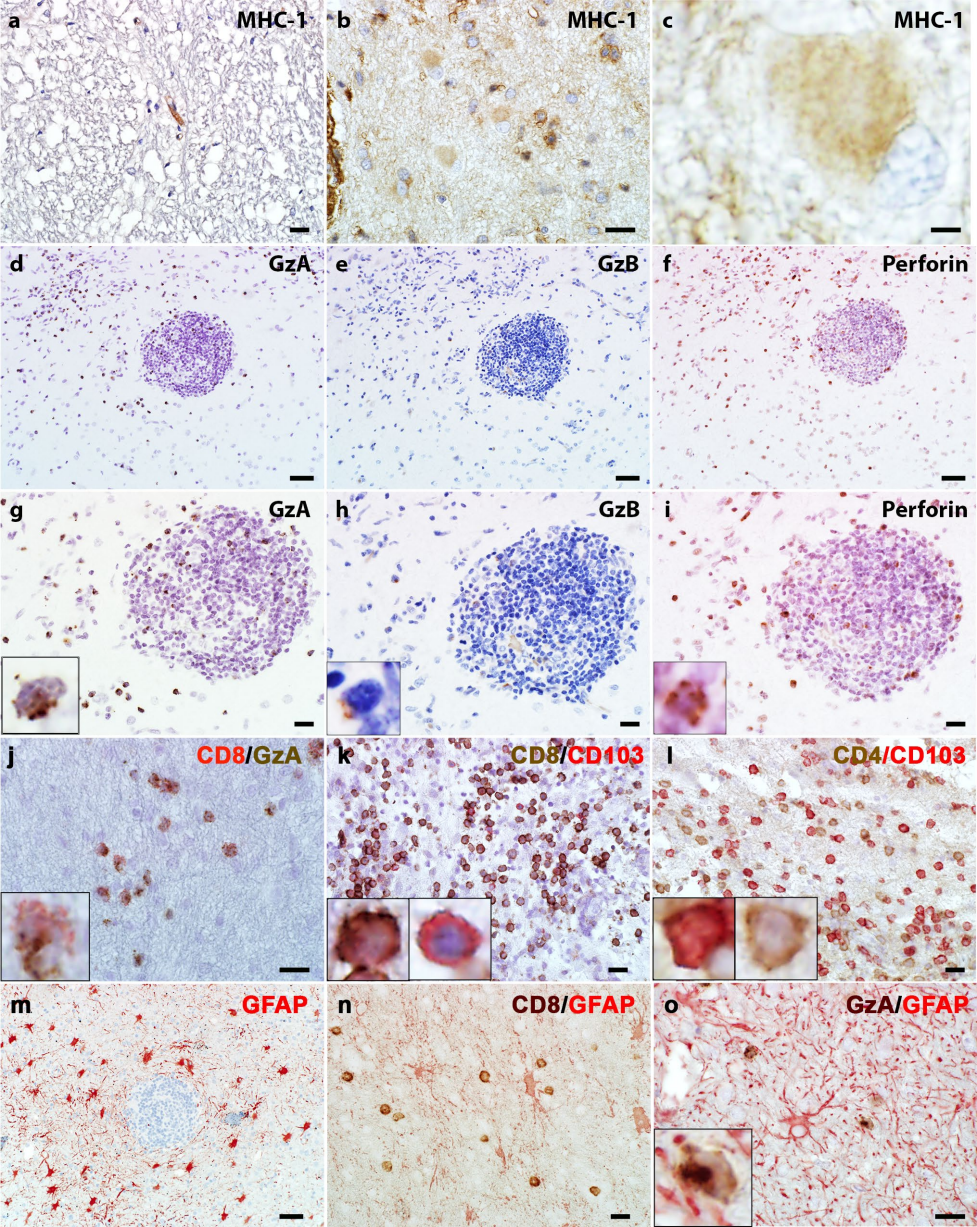
MHC-1 expression in reactive astrocytes and cytotoxic T lymphocytes is present in the CNS of GFAP autoimmunity **(a)** The normal CNS shows limited expression of MHC-1 in the endothelial cells, which is not detectable in astrocytes. **(b)** The brain biopsy of a patient with anti-GFAP autoantibodies shows increased MHC-1 immunoreactivity in both cytoplasm and cell processes. Note some infiltrating leukocytes show strong membrane MHC-1 immunoreactivities. **(c)** The magnified view of **(b)** shows increased MHC-1 signals in cytoplasm and processes in a hypertrophic reactive astrocyte. **(d-f)** and magnified views **(g-i)** show cytotoxic T lymphocyte markers on consecutive tissue sections: more T lymphocytes show granzyme A (GzA) positivity **(d, g)** than granzyme B (GzB) **(e, h)**. Perforin-positive cells **(f, i)** are numerically similar to granzyme A. **(j)** Double immunostain shows a fraction of lymphocytes positive for CD8 (red) and granzyme A (GzA, brown). **(k)** Many CD8-positive T cells (brown) are also positive for CD103 (red). The left inset shows a magnified view of a CD8^+^CD103^+^ cell. The right inset shows a CD8^−^CD103^+^ cell. **(l)** A few CD4 lymphocytes are also positive for CD103. The left inset shows a CD4^+^CD103^+^ cell. The right inset shows a CD4^+^CD103^−^ cell. **(m)** GFAP stain shows diffuse hypertrophic astrocytes around an inflamed vessel. **(n)** Double immunostain for CD8 (brown) and GFAP (red) indicates the close contact between astrocytes and CD8^+^ lymphocytes. **(o)** Double stains for granzyme A (GzA, brown) and GFAP (red) show a cytotoxic T lymphocyte polarizing the granzyme A granules towards the astrocyte process (inset). Scale bars **a, b, g-l, n, o** 20 µm; **c** 3 µm; **d-f, m** 50 µm. *CD* cluster of differentiation, *GFAP* glial fibrillary acidic protein, *GzA* granzyme A, *GzB* granzyme B, *MHC-1* major histocompatibility complex class I

All biopsy cases (n = 9) but none of the controls (n = 3) showed MHC class I immunoreactivity in reactive astrocytes (Fig. 7a-c). CD8^+^ lymphocytes co-expressed cytotoxic markers, namely perforin (7/8), granzyme A (8/8), and granzyme B (8/8) (Fig. 7d–j). Granzyme A positive cells were more abundant than granzyme B positive cells. Further analyses revealed the co-expression of CD103, a marker for tissue resident memory cells, in some of the infiltrating CD4^+^ and CD8^+^ lymphocytes. The majority of CD103 positive cells were CD8^+^ lymphocytes (Fig. 7k, l). We performed double labeling for cytotoxic T cell markers and GFAP in 4 biopsy cases and found a close apposition of T lymphocytes, with polarized location of granzyme A/B and perforin granules towards the GFAP positive astrocytes (4/4) (Fig. 7m-o).

### Astrocytic reaction in GFAP autoimmunity

**Fig. 8:**
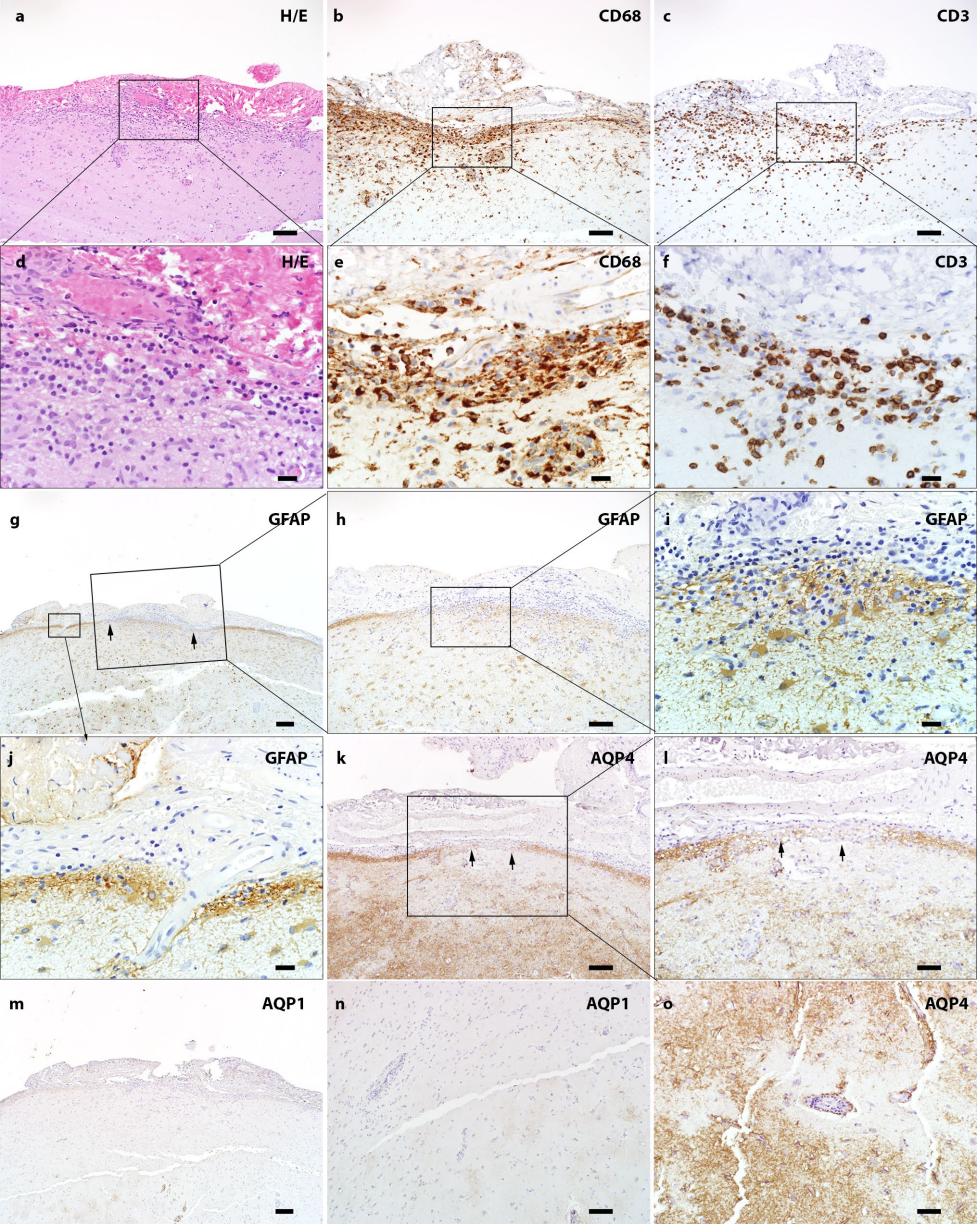
Subpial pathology and parenchymal aquaporin loss in GFAP autoimmunity **(a)** H&E staining shows hypercellularity in the leptomeninges and subpial parenchyma. Increased collagen is noted in the leptomeninges (magnified view in panel **d**). **(b)** CD68 on the consecutive section highlights marked macrophages/microglia in both meninges and subpial parenchyma (high power view in panel **e**). **(c)** Infiltration of T lymphocytes in meninges and towards the deep parenchyma is seen in the same region (magnified view in panel **f**). **(g-j)** GFAP staining indicates alteration of glia limitans in the inflamed area. **(h)** Decreased GFAP along pial surface. **(i)** The high-power view highlights discontinuous glia limitans and some adjacent hypertrophic astrocytes. **(j)** The adjacent non-inflamed region shows intact glia limitans. **(k)** Immunohistochemistry indicates decreased pial AQP4 immunoreactivity in the highly inflamed region (high power view in **l**). **(m)** Decreased pial AQP1 is also noted. **(n)** Cortical perivascular loss of AQP1 immunoreactivity. **(o)** Perivascular decreased AQP4 immunoreactivity. Scale bars **a-c** 100 µm; **g, m** 200 µm; **l, n, o** 50 µm; **h, k** 100 µm; **d-f, i, j** 20 µm. *AQP1* aquaporin 1, *AQP4* aquaporin 4, *CD* cluster of differentiation, *GFAP* glial fibrillary acidic protein, *H&E* hematoxylin & eosin

The astrocytes showed hypertrophic morphology at sites of inflammation and in the surrounding area (9/9). We did not observe remarkable astrocytic lysis either in the cortex nor in the white matter. However, the subpial glia limitans occasionally presented with focal discontinuity and alteration of astrocytic arrangement (Fig. 8a–m).

To further characterize the astrocytic pathology, we evaluated AQP1 and AQP4 immunoreactivity (Fig. 8k–o). Variable changes of AQP1 and AQP4 were found in biopsied CNS samples compared with normal controls (Table 2) reflecting the astrocytic reactions to stress within the context of GFAP autoimmunity.

**Table 2:** Changes of AQP1 and AQP4 immunoreactivities in the CNS of GFAP autoimmunity

	Normal control	GFAP Autoimmunity (Fig. 8k–o)	
		Cortex	White matter
AQP1	Cortex < white matter	Normal^a^ (3/5) Increased (1/5) Focally decreased (1/5)	Increased (4/6) Increased and decreased in different locations (2/6)
AQP4	Cortex > white matter	Normal (1/4) Increased (2/4) Focal perivascular and subpial decreased (1/4)	Increased (5/5)

^a^Normal, increased and decreased, mean the immunoreactivity compared with normal control tissue

*AQP1* aquaporin1, *AQP4* aquaporin 4, *GFAP* glial fibrillary acidic protein

### Canine autopsy-clinical and neuropathological features in pug dog encephalitis

**Fig. 9:**
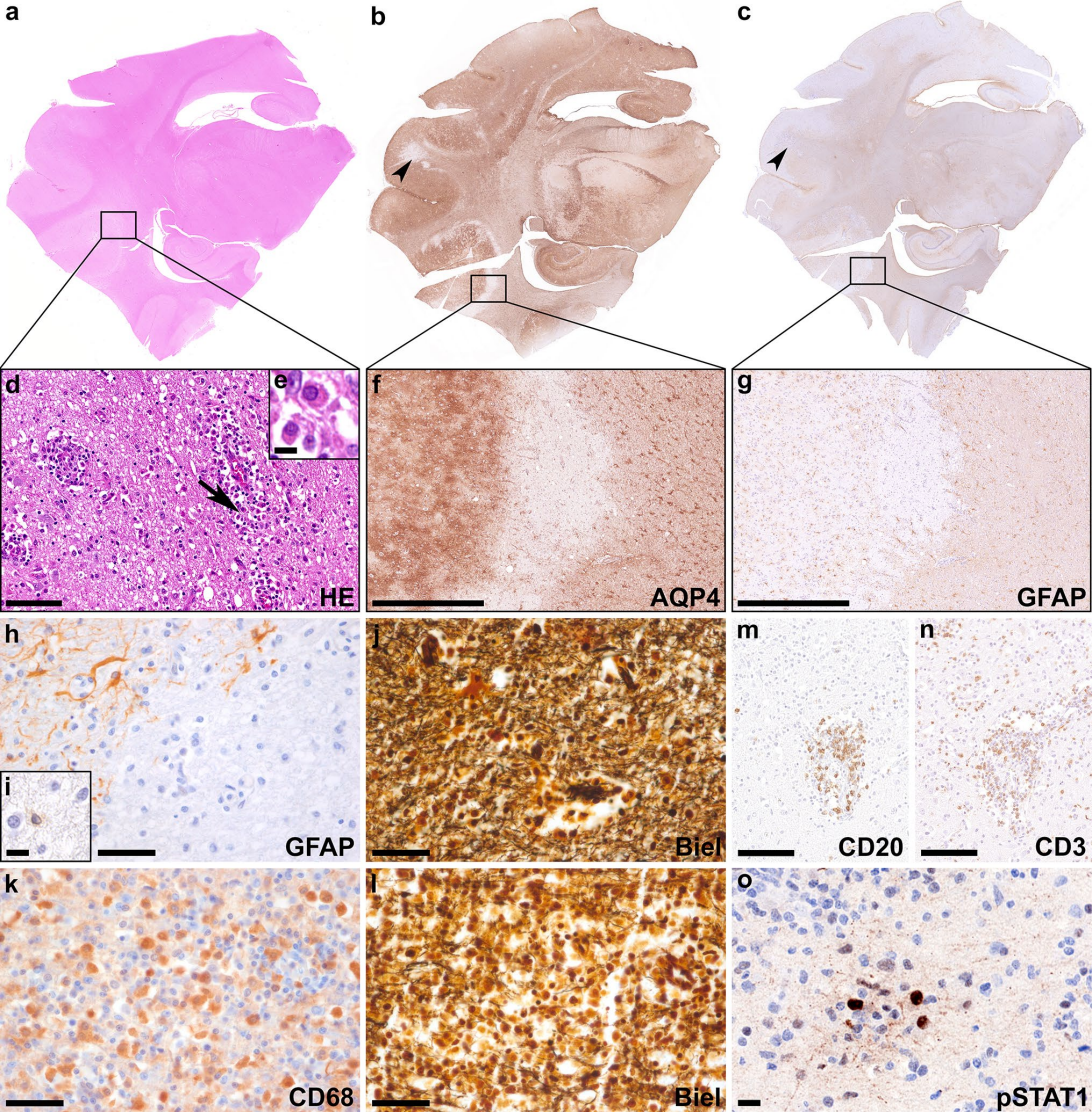
Neuropathological findings in a canine autopsy case with anti-GFAP autoantibodies Overview stainings (H&E **(a)**; AQP4 **(b)**; GFAP **(c)**) of the same area of a canine temporal brain section including the hippocampus. H&E staining **(d)** shows edema, reactive gliosis, neutrophilic granulocytes and mitoses and perivascular inflammatory infiltrates mainly consist of lymphocytes and plasma cells (arrow in **d** enlarged in inset **e**). In some parts of the lesions AQP4 **(f)** and GFAP **(g, h)** are selectively lost **(h)** with a few apoptotic astrocytes remaining within the lesion **(i),** while axons are relatively well preserved **(j**; Biel**)**. Other parts of the lesions showed focal cystic necrosis with abundant CD68 positive macrophages **(k)** and loss of axons **(l;** Biel**)**. Immunohistochemistry reveals some CD20^+^ B cells **(m)** around vessels. Inflammation is characterized by an abundant number of perivascular and parenchymal CD3^+^ T cells **(n)**. pSTAT1 is mainly upregulated in lymphocytes **(o)**. Scale bars **d, m, n** 50 µm; **f, g** 500 µm; **e, i, o** 10 µm; **h, j-l** 25 µm. *AQP4* aquaporin 4, *Biel* Bielschowsky, *CD* cluster of differentiation, *GFAP* glial fibrillary acidic protein, *H&E* hematoxylin & eosin, *pSTAT1* phosphorylated signal transducer and activator of transcription 1

A five-year-old, non-neutered female Shih Tzu dog was presented to the emergency services of the Institute of Internal Veterinary Medicine, Veterinary University of Vienna, Austria. Two days earlier, the dog had a convulsion and the owners reported that she did not want to go outside for a few days. Clinical examination was carried out, and focal and generalized epileptic seizures were diagnosed. The dog was not able to walk, and she was vocalizing and defecating. Hematologic and chemical parameters were unremarkable. Despite immediate antiepileptic and symptomatic medical treatment, the dog died the next day. Necropsy was performed and histological examination of the brain revealed a marked lymphocytic/lymphomonocytic meningoencephalitis with a focal necrotizing character, particularly in the temporoparietal cerebral cortex (NME) (Fig. 9a–e). Parts of the inflammatory lesions were characterized by a selective loss of astrocytes with loss of AQP4 (Fig. 9f) and GFAP (Fig. 9g, h) with a few apoptotic astrocytes remaining within the lesion (Fig. 9i), while axons were relatively well preserved (Fig. 9j). Other parts of the lesions showed focal cystic necrosis with abundant macrophages (Fig. 9k) and loss of axons (Fig. 9l). Immunohistochemistry revealed some perivascular CD20^+^ B cells (Fig. 9m), as well as abundant perivascular and parenchymal CD3^+^ T cells (Fig. 9n). Microglia were strongly activated around vessels and in the brain parenchyma (data not shown). The interferon signaling marker pSTAT1 was mainly upregulated in lymphocytes (Fig. 9o). Anti-GFAP autoantibodies were detected post-mortem in CSF using an in house TBA and a fixed CBA (Supplementary Fig. 1; Online Resource 1). Based on these results, the diagnosis of pug dog encephalitis was made.

## Discussion

In the present study we describe the neuropathological and inflammatory features of subacute anti-GFAP meningoencephalomyelitis in two human autopsies and nine biopsies and observed a lymphocytic phenotype in the majority of patients, one autopsy case revealed a prominent granulomatous phenotype. Within these phenotypes we found evidence for a T cell-mediated immune reaction against astrocytes and activation and deposition of C4d of the complement system along astrocytic membranes. Inflammatory infiltrates were composed of B and T cells, including tissue resident memory T cells. An acute evolution in a pug dog encephalitis allowed us to provide insight into an early phase of GFAP autoimmunity in a canine autopsy case.

Neuropathological reports of anti-GFAP meningoencephalomyelitis in humans are rare [11, 17, 19, 23, 39, 48, 52]. These studies described lymphocytic inflammation and reactive gliosis but no loss or decrease in GFAP immunostaining, fragmentation or phagocytosis of astrocytes. These features would be consistent with a lack of an effector role for anti-GFAP autoantibodies, which would be predicted by GFAP’s cytoplasmic location inaccessible to IgG [48]. In our human case series, an obvious astrocytic damage was also absent in H&E and GFAP-staining. However, we found an upregulation of MHC class I and granzyme and perforin expressing CD8^+^ T cells in close apposition to astrocytes, which suggests that cytotoxic T cell-mediated immune reaction is present in GFAP autoimmunity. In addition, we found a strong activation of the complement component C4d that specifically labeled the surface of astrocytes.

The reason for the lack of obvious astrocytic damage in GFAP autoimmunity (which does not necessarily mean lack of dysfunction) might be a time-dependent factor. In a rodent model of acute monophasic GFAP encephalomyelitis, brain inflammation starts two days after T cell transfer and reaches a peak at day five to seven, after which the animals fully recover [5, 36]. Inflammation is characterized by profound T cell infiltration, activation of resident microglia, in the near absence of recruitment of systemic myeloid cells. Focal lymphocyte infiltration is associated with a close contact of CD8^+^ T lymphocytes with MHC class I expressing astrocytes and with astrocyte apoptosis. This results in perivascular loss of astrocytes and their cell processes at the glia limitans. However, astrocytes are replenished within a few days after the peak of the acute inflammatory attack and the animals recover without clinical deficits or permanent neuropathological lesions [5]. In human autopsy and biopsy tissues the regenerating astrocytes might be reflected by numerous Ki-67 positive astrocytes that are evident throughout the gray and white matter.

Our canine autopsy case of pug dog encephalitis, which was obtained three days after disease onset, i.e., during the peak of disease, resembles the histopathological findings of the acute monophasic encephalomyelitis in rodents. It showed a marked meningoencephalitis with astrocytic damage characterized by loss of GFAP and AQP4 within the lesions. We could not evaluate C4d because the commercial antibody is human specific and does not cross react with the canine epitope.

All our human cases had a subacute evolution and a much longer disease duration in comparison to the above discussed pug dog encephalitis. In this series, we found two main morphological phenotypes, a lymphocytic and a granulomatous inflammatory form. However, and as described in an experimental GFAP-T cell receptor (GFAP-TCR) transgenic mouse model, the coexistence of variable phenotypes in anti-GFAP meningoencephalomyelitis is possible at the CNS level. The authors observed two distinct CNS autoimmune phenotypes depending on the initiating trigger that lead to the activation of auto-reactive CD8 effector T cells [36]: (1) Vaccination with the GFAP protein resulted in direct activation of GFAP-specific CD8 T effector cells, which infiltrated the meninges and the perivascular space within the gray and white matter, causing localized necrosis and apoptosis. The mice developed a rapid and acute neurological phenotype with ataxia, spasticity, and lethargy. (2) In more chronic lesions CD103^+^ tissue resident memory T cells persisted within the tissue and may propagate the inflammatory response with activation of innate immune responses, resulting in massive necrosis and apoptosis. These mice showed a more chronic clinical phenotype that started between 30 and 60 days after birth and gradually increased in severity, associated with a gradual increase of inflammation in the brain and spinal cord. Only a small percentage of the CD8^+^ T cells were fully activated effector cells, while a large proportion of the cells acquired the phenotype of tissue resident memory cells. At focal sites of dense inflammatory infiltration astrocytes were lost and apoptosis of some MHC class I expressing astrocytes was present at the edge of the lesions. Thus, the different pathologies seen in human GFAP autoimmunity may reflect different stages of lesion development. Alternatively, the lymphocytic and granulomatous phenotypes in human GFAP autoimmunity may reflect different triggers or the influence of specific genetic risk variants.

One of our autopsy cases showed bilateral trigeminal nerve enhancement in the MRI, in addition neuropathological workup revealed inflammation in the epi-, peri- and endoneurium of the spinal nerve roots, compatible with a polyradiculitis. The involvement of the peripheral nervous system in GFAP autoimmunity has previously been reported [31]. In a series of 103 patients 24% showed an involvement of the peripheral nervous system, mostly affecting cranial nerves and/or lower limb radiculopathy [41]. Although we could not detect specific GFAP immunoreactivity in the nerve roots in our case, a low level (below immunohistochemical detectability) of GFAP expression in Schwann cells and/or fibroblasts could be involved in triggering neuritis in GFAP autoimmunity [16, 41].

Interestingly, the strong and widespread labeling of C4d along astrocytic membranes including processes was only detectable in cases with a lymphocytic phenotype. The C4d labeling was specific for GFAP autoimmunity and was not detectable on astrocytes in GFAP-antibody-negative cases of anti-Ma2-associated encephalitis (n = 1), anti-neuronal autoimmune encephalitis (anti-NMDAR n = 3, anti-AMPAR encephalitis n = 2), Alzheimer’s disease (n = 2), MOG antibody associated disorders (n = 1), stroke (n = 1) and healthy controls (n = 6). No other signs of complement activation on astrocytes, such as the deposition of C1q and C5b-9 were detected in GFAP autoimmunity.

The pathogenic role of C4d in GFAP autoimmunity is unclear. C4d is a cleavage product of the complement protein C4 and is generated in the process of complement activation, which, after splicing, is covalently bound to the tissue and remains close to the site of activation [6].

Recently it has been demonstrated that activated astrocytes in multiple sclerosis lesions show a significant upregulation of complement complex activators, receptors and complement components [1], but as it has also been shown by others before, this complement activation was mainly reflected by increased deposition of early complement components such as C1q or C3 [28, 29]. Alternatively, C4d might be directly activated by other factors such as neoplasias [7], neurodegeneration like Alzheimer’s disease [51], or induced in astrocytes by activation or exposure to pro-inflammatory stimuli, such as interferon-gamma (IFNγ) [34, 44]. Subsequently, it is bound to the cell surface of astrocytes e.g. via NRP1 [4, 21], which is also induced in the course of activation [18]. In our case series, C4d and NRP1 positive astrocytes may reflect the focal interaction of astrocytes with activated CD8^+^ T cells in the process of antigen presentation with the secretion of IFNγ in the cleft of the immunological synapse.

In comparison, the multinucleated giant cell formation in the granulomatous phenotype of anti-GFAP meningoencephalomyelitis might be caused via the continuous induction of inflammatory cytokines released by the infiltrating leukocytes and reactive resident glial cells [27]. Within the granulomas, pSTAT1 was strongly upregulated in lymphocytes, glial cells, and macrophages. In addition, we found a significantly higher amount of perivascular and parenchymal CD103^+^ tissue resident memory T cells in the cerebellum and brainstem compared to the autopsy with lymphocytic phenotype. The granulomatous lesion formation might thus be a later phenomenon compared with the lymphocytic infiltration. Importantly, GFAP autoimmunity can clinically and pathologically also mimic tuberculous meningoencephalitis or other granulomatous inflammations and should therefore be considered as differential diagnosis [33]. Interestingly, two patients with systemic sarcoidosis were recently described to be anti-GFAP autoantibody positive (published as abstract in Neurology Dec 05, 2022; 99 (23 Suppl 2) DOI: https://doi.org/10.1212/01.wnl.0000903452.01952.40). However, in our autopsy case no underlying systemic granulomatous disease was found.

So far, no genetic predisposition for anti-GFAP meningoencephalomyelitis has been identified. In a recent study, HLA typing of 26 patients with GFAP autoimmunity did not reveal significant differences in carrier frequencies between patients and controls for class I (A, B, C) and class II (DRB1, DQB1, DQA1, and DPB1) genes [13], however in dogs anti-GFAP meningoencephalomyelitis is limited to specific breeds [3, 14, 15, 32, 35, 37] what likely supports the existence of some genetic susceptibility factors. Future genome wide association studies will be necessary to elucidate possible genetic risk factors in humans that may predispose to the development of anti-GFAP meningoencephalomyelitis.

## Conclusion

In summary, we have identified both lymphocytic and granulomatous inflammatory phenotypes in subacute human anti-GFAP meningoencephalomyelitis and provide a detailed analysis of potential immune pathomechanisms and astrocytic reaction that might be stage-dependent. While in subacute/chronic disease stages an overt astrocytic damage was absent, the MHC class I upregulation along with CD8^+^/perforin^+^/granzyme A/B^+^ T cells attached to astrocytes suggest that cytotoxic T cell-mediated immune reaction is present in GFAP autoimmunity. In addition, complement C4d activation might play a role in GFAP autoimmunity, either as cause or consequence of astrocytic reactivity. Future studies will be necessary to investigate the possible impact of these mechanisms on the clinical disease course and therapeutic consequences.

### Supplementary Information

Below is the link to the electronic supplementary material.

Supplementary file1 (DOCX 13249 KB)

## Data Availability

Data can be made available from the corresponding authors on reasonable request and after approval from the ethics review board at the Medical University of Vienna.
