# Supplementary material for: New insights into neuropathology and pathogenesis of autoimmune glial fibrillary acidic protein meningoencephalomyelitis
Source: Acta Neuropathol. 2024 Feb 3;147(1):31. doi: 10.1007/s00401-023-02678-7 (PMC10838242; doi:10.1007/s00401-023-02678-7)

**SUPPLEMENTARY FIGURE LEGEND**

**Supplementary Fig. 1 Tissue-based assay (TBA) and cell-based assay (CBA) of the canine autopsy case with anti-GFAP autoantibodies** Incubation of CSF of a dog with pug dog encephalitis on rat TBA reveals positively stained astrocytes (arrow in **a** enlarged in inset) in the hippocampus **(a)** and marked radial glial processes (black rectangle in **b** enlarged in inset) in the cerebellum **(b)** in comparison to a control dog without astrocyte staining **(c, d)**. GFAPα antibodies were confirmed in CSF by a fixed CBA **(e-g;** GFP-tag of GFAPα transfected cells green, patient CSF red, merge yellow**)**, while CSF of a control dog remained negative **(h-j)**. Scale bar **a-d** 250 µm; **inset a, b** 25 µm; **e-j** 50 µm.

α = alpha, GFAP = glial fibrillary acidic protein, GFP = green fluorescent protein, IgG = immunoglobulin type G, neg. = negative, pos. = positive.


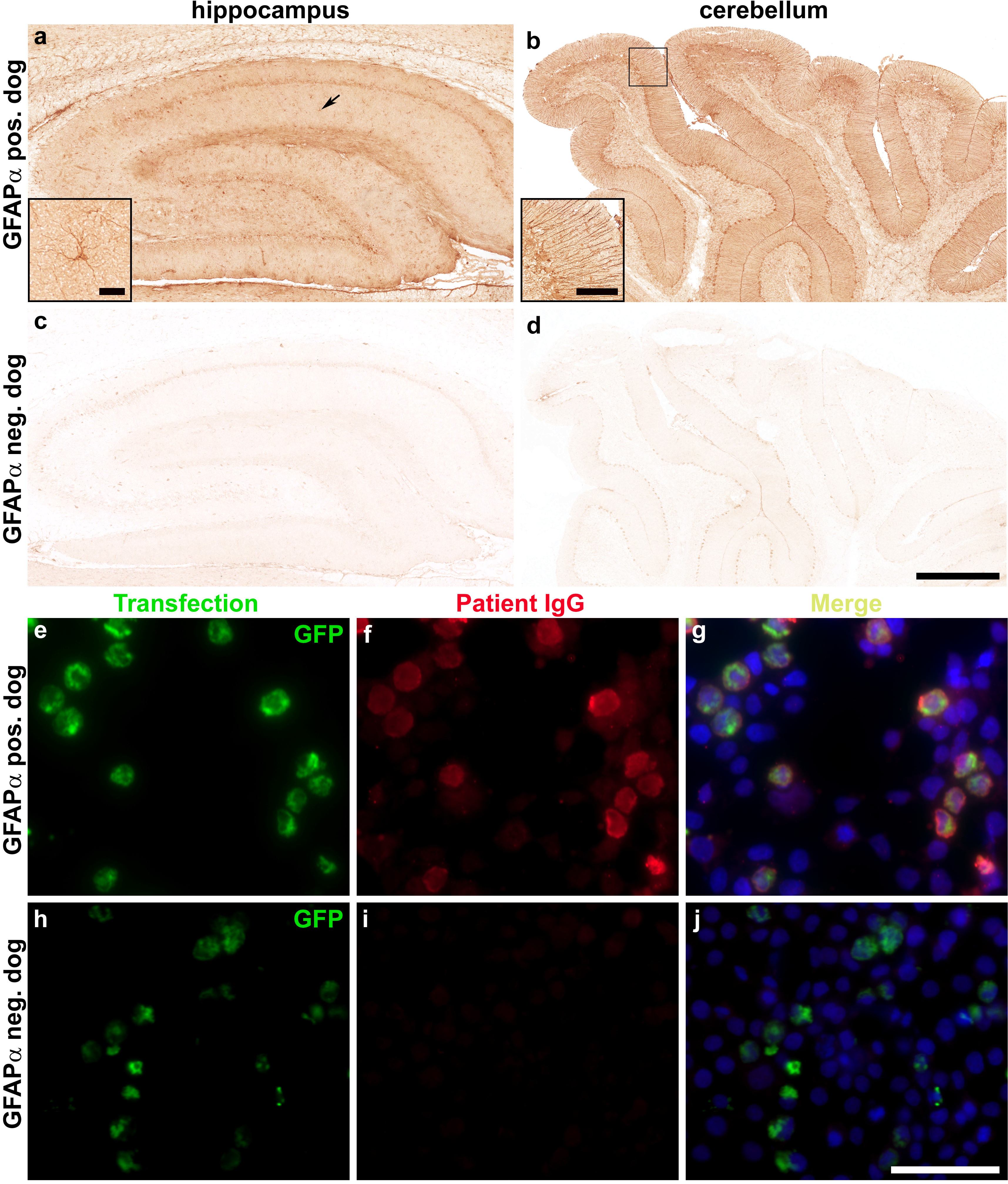

Supplement: Supplementary file 1 — Supplementary file1 (DOCX 13249 KB) [file 401_2023_2678_MOESM1_ESM.docx]
